# Low frequency oscillations reflect neurovascular coupling and disappear after cerebral death

**DOI:** 10.1038/s41598-024-61819-4

**Published:** 2024-05-17

**Authors:** Sven Schulthess, Susanne Friedl, Gagan Narula, Giovanna Brandi, Jan Folkard Willms, Emanuela Keller, Giulio Bicciato

**Affiliations:** 1 Neurocritical Care Unit, Department of Neurosurgery, Institute of Intensive Care Medicine, University Hospital, University of Zurich, 8091 Zurich, Switzerland; 2 Department of Neurology, University Hospital Zurich, University of Zurich, 8091 Zurich, Switzerland

**Keywords:** Prognostic markers, Neurology, Brain injuries, Cerebrovascular disorders, Stroke, Auditory system, Neuro-vascular interactions, Somatosensory system

## Abstract

Spectrum power analysis in the low frequency oscillations (LFO) region of functional near infrared spectroscopy (fNIRS) is a promising method to deliver information about brain activation and therefore might be used for prognostication in patients with disorders of consciousness in the neurocritical care unit alongside with established methods. In this study, we measure the cortical hemodynamic response measured by fNIRS in the LFO region following auditory and somatosensory stimulation in healthy subjects. The significant hemodynamic reaction in the contralateral hemisphere correlation with the physiologic electric response suggests neurovascular coupling. In addition, we investigate power spectrum changes in steady state measurements of cerebral death patients and healthy subjects in the LFO region, the frequency of the heartbeat and respiration. The spectral power within the LFO region was lower in the patients with cerebral death compared to the healthy subjects, whereas there were no differences in spectral power for physiological activities such as heartbeat and respiration rate. This finding indicates the cerebral origin of our low frequency measurements. Therefore, LFO measurements are a potential method to detect brain activation in patients with disorders of consciousness and cerebral death. However, further studies in patients are needed to investigate its potential clinical use.

## Introduction

Outcome prognostication in patients with acute disorders of consciousness (DoC) has an impact on fundamental choices about therapy continuation or redirection of care to palliation^1^. Prognostic tests should be 100% specific for prediction of bad outcome, thus avoiding self-fulfilling prophecies. Current established methods for outcome prognostications include clinical evaluation, neuroimaging, electroencephalography (EEG) and evoked potentials. In contrary the role of other techniques such as functional neuroimaging (e.g. functional magnetic resonance, positron emission tomography or transcranial magnetic stimulation) and neurophysiological techniques including functional near-infrared spectroscopy is still debated^2–10^. Technology innovation in the field of neurophysiology is likely to enhance our ability to measure clinically covered brain activity for diagnostic and prognostic purposes^11–15^. Detection of brain activation in clinically unresponsive patients with traumatic brain injury through EEG has been already associated with a better outcome^16^. An expanded and multimodal diagnostic toolbox may achieve higher accuracy in the measure of brain activity and thus increase accuracy of prognostication in patients with DoC^15,17^. Functional near-infrared spectroscopy (fNIRS) offers portability, low cost, and non-invasiveness; however it has not yet been validated as a diagnostic or prognostic tool^18^. The limited understanding of the neuropathological significance of the hemodynamic activity measured by fNIRS represents a main pitfall preventing its integration among clinical and electrophysiological examinations. Hence, a better knowledge of the neurophysiology and neuropathology may be key to make fNIRS enter the diagnostic toolbox of the clinicians. In our prior research, we demonstrated that low frequency oscillations (LFO) in the left prefrontal areas exhibit increased amplitude in healthy subjects after auditory stimulation with music, showing diminished reactivity in patients with reduced consciousness^19,20^. Auditory and somatosensory evoked potentials (AEP and SEP) are already established and standardized measurements in outcome prognostication in patients with DoC. They have the advantage of being less susceptible to the effects of patient sedation than prefrontal reaction to music stimulation^21^. By measuring cerebral hemodynamic response by fNIRS additionally to the electric response of AEP and SEP the accuracy of these assessments may be enhanced. Our first objective in this study is therefore to investigate LFO amplitude changes in healthy subjects following somatosensory or auditory stimuli. To further understand whether LFO reflects a specific neurogenic cerebral hemodynamic activity or a systemic sympathetic activation, we additionaly investigate LFO spectral power along with the power spectrum of heart beat and respiration frequencies in patients with the clinical diagnosis of cerebral death and comparing it to healthy subjects during resting state measurements.

## Methods

### Participants

The study was performed at the Neurocritical Care Unit (NCCU) of the University Hospital Zurich. The study included healthy subjects for fNIRS measurements with evoked potentials and as a control group for resting state measurements. Inclusion criteria were full consciousness (GCS = 15) and an age of 18 years or more. Excluded were subjects with any history of cerebral or brainstem diseases, previous auditory complaints or any ear disease, no response detectable at Erb’s point in SEP (e.g. due to peripheral nerve lesions, or edema). Written informed consent was given from all participants. For our resting state measurements we included patients from the NCCU with the diagnosis of cerebral death, defined by the neurological examination with Glasgow Coma Scale (GCS) 3 without sedation since at least 24 h, dilated and unreactive pupils, and absent brainstem reflexes. Exclusion criteria was hemodynamic instability according to the judgement of the treating physician to avoid concurring interventions during the measurement. Written informed consent was given by legal representatives, as all patients were incapable of judgment. The study was approved by the local ethics committee (Kantonale Ethikkomission Zürich, KEK-Nr.2019-02192) and performed in accordance with the Declaration of Helsinki.

### Demographics

**Figure 1:**
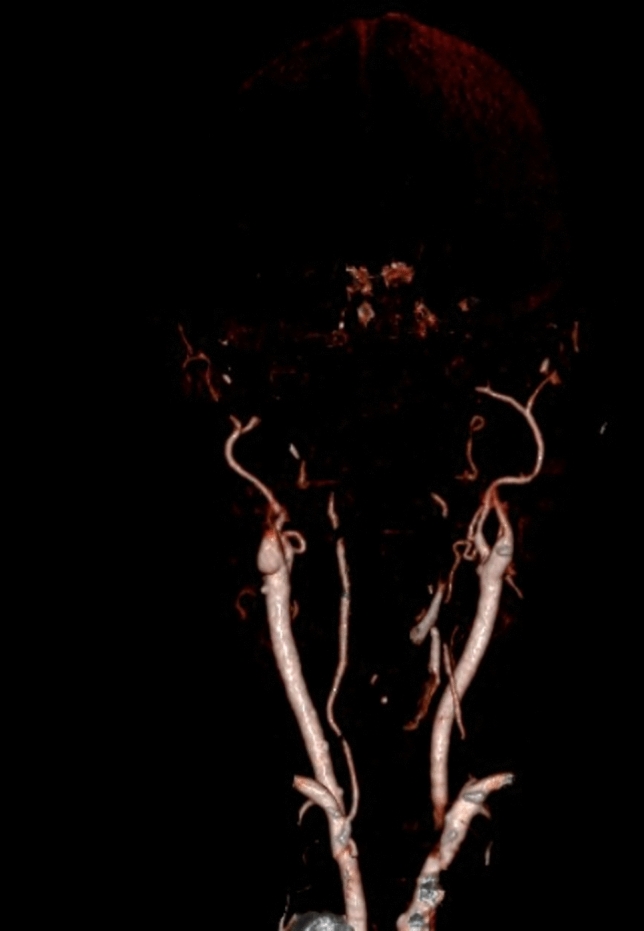
Cerebral computed tomography angiography of Subject 2. Reconstruction showing a typical finding compatible with cerebral death. No contrast filling of both intern carotid arteries after the petrous segment. Both vertebral arteries present no filling after the V3 segment. The extern carotid arteries are bilaterally perfused.

Seven patients of the Neurocritical Care Unit (NCCU) of the University Hospital Zurich with the clinical diagnosis of cerebral death were included. Demographic data of the patients are shown in Table 1. In five out of seven patients a computed tomography–angiography (CTA) could additionally demonstrate a cessation of cerebral circulation (Fig. 1), in two patients no CTA was performed. All patients with cerebral death were mechanically ventilated and hemodynamically stable. Underlying pathology for all the patients was aneurysmal subarachnoid hemorrhage and the supposed mechanism of cerebral death was intracranial hypertension with consecutive transforaminal herniation. Mean age [SD] of the 7 patients was 56.7 years (SD ± 16.6). Three patients were men and four were women. 6 healthy subjects were enrolled (2 females, 4 males). Mean age of the healthy subjects was 41.4 years (SD ± 12.6). The age difference in between the patients and healthy subjects was not significant (p = 0.08). Data of the healthy subjects have been already published in previous studies^19,20^.

**Table 1:** Demographics of patients with clinical diagnosis of cerebral death.

	Age (years)	Sex	Pathology	Mechanism of cerebral death	Diagnosis	Days since acute brain injury
Patient 1	59	m	aSAB	Increased intracranial pressure	Clinical and CTA	1
Patient 2	74	f			Clinical and CTA	2
Patient 3	75	m			Clinical	21
Patient 4	59	f			Clinical and CTA	1
Patient 5	61	f			Clinical	1
Patient 6	36	m			Clinical and CTA	1
Patient 7	33	f			Clinical and CTA	2

*m* masculine, *f* feminine, *CTA* cerebral computed tomography angiography, *aSAB* aneurysmal subarachnoid hemorrhage.

### Experimental protocol

#### SEP-fNIRS and AEP-fNIRS measurements

**Figure 2:**
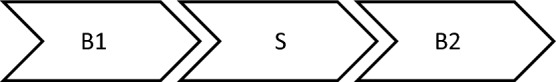
Experimental protocol. Functional near-infrared spectroscopy measurements to sensory stimulation were preceeded and followed by a baseline measurement. *B1* first baseline block, *S* sensory stimulation block (either AEP or SEP), *B2* second baseline block. Each block lasting about 3 min.

SEP and AEP were conducted successively on the left and right side in all healthy participants with a simultaneous fNIRS measurement of both hemispheres. The measurements were preceded and followed by a baseline block without any stimuli, each lasting three minutes as shown in Fig. 2.

#### Resting state recordings

For each participant (patients and healthy subjects) a resting-state fNIRS recording of 5 min was acquired. External interactions with the subject during the recording was not allowed (e.g. no nursing intervention or manipulation of the ventilation setting).

### FNIRS measurement

**Figure 3:**
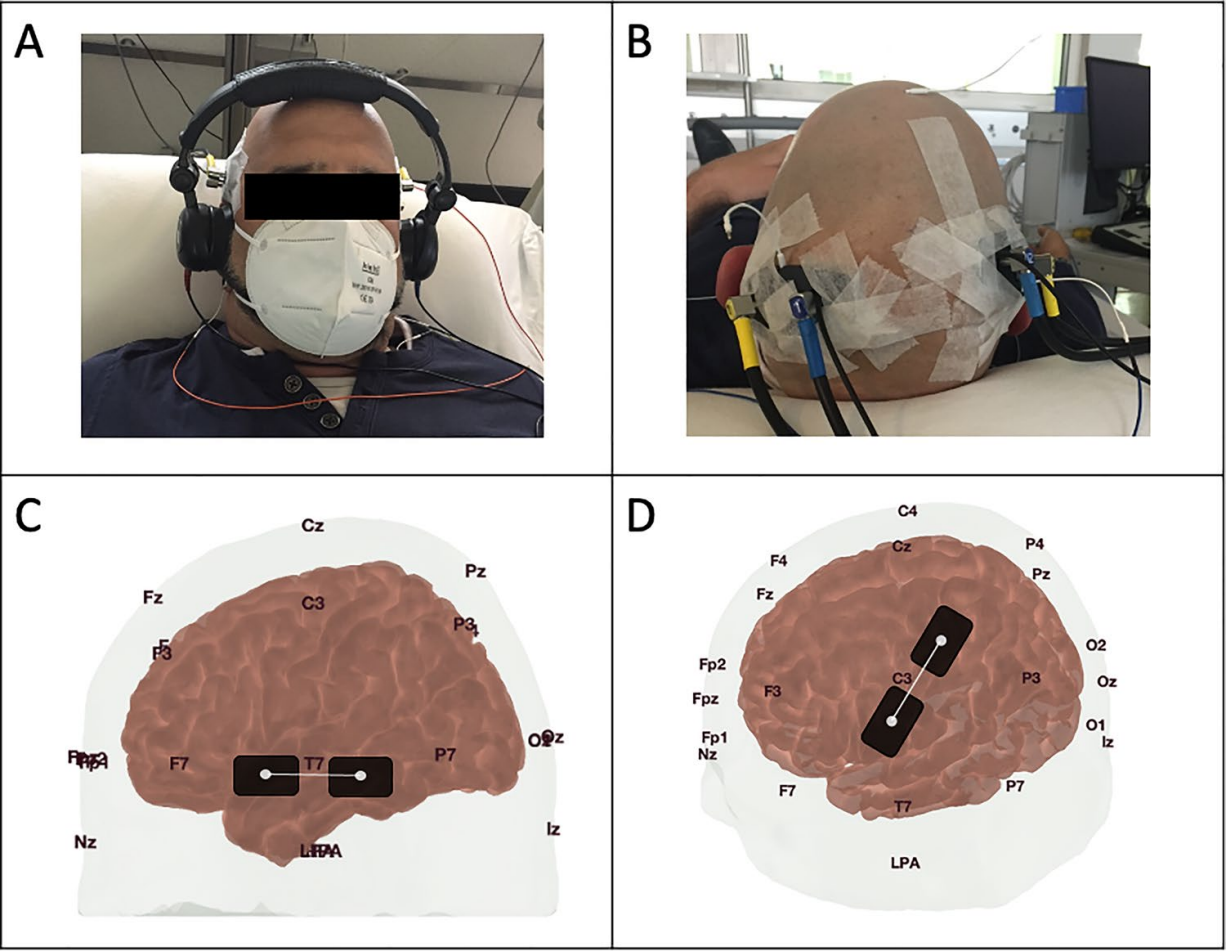
Visualization of the optode placement. The 10–20-EEG System was used as reference points for the optode placement. Regarding the photos in (**A,B**) informed consent for the publication in an open access journal was given from the subject. (**A**) Healthy subject with the optode placement during AEP measurement at the T7/8 position. (**B**) Healthy subject with the optode placement during SEP measurement 2-3 cm behind the C3/4 position. (**C**) Head model of the open access atlas viewer software to demonstrate the optode placement on the left cortex during a AEP measurement. (**D**) Head model of the open access atlas viewer software to demonstrate the optode placement on the left cortex during a SEP measurement^58^.

Data acquisition and analysis were performed with OXYMON Mk III and Oxysoft (version 3.0, 103.3, Artinis Medical Systems B.V., Elst, The Netherlands). Resting-state measurements were carried out by applying two optodes on the forehead bilaterally as already described in our previous works^19^. During AEP the optodes were applied on the temporal lobe bilaterally (T7 and T8 position of 10–20 EEG System). During SEP the optodes were applied in correspondence of the primary sensory cortex on the parietal lobe about 2–3 cm behind C3 and C4, respectively^22^. Optodes positions during the AEP and SEP measurements are depicted in Fig. 3. Interoptode distance i.e. the distance between the main light source and the light detector was 35 mm^23^. An auxiliary short channel with interoptode distance of 10 mm was applied in the fNIRS SEP/AEP measurement but not in the resting state recording. The optical signal of the short channel was subtracted from the main signal to reduce the effect of the superficial signal originating from the scalp^24–27^. The sampling frequency was 25 Hz. The differential pathlength factor (DPF) was 6. Oxyhemoglobin (O_2_Hb) was automatically computed according to the modified Lambert–Beer law by Oxysoft software.

### SEP/AEP measurements

SEP and AEP in healthy subjects were performed, using Dantec Keypoint G4 EMG/NLG/EP workstation (Neurolite). Both measurements were conducted according to the clinical practice in our center. In the SEP measurement stimulation of the ulnaris nerve using sterile subcutaneous needle electrodes (10 mm × 0.30 mm Natus) was performed. Visible motor response of the adductor pollicis muscle was observed as a marker of adequate stimulation intensity. Stimulation frequency was 3.1 Hz with each stimulus lasting 0.2 ms. For both sides two consecutive measurement rounds with each consisting > 300 stimulation responses were conducted. For recording subcutaneous needle electrodes were positioned above the somatosensory cortex according to the 10–20 EEG System and Erb’s point^21^. SEP were rated according to latency and amplitude of the collected cortical potentials, respectively. Latency was normalized for body height. Limits were determined according to laboratory-determined reference values. In the AEP measurements each patient was stimulated with a click sound with a stimulation frequency of 9.8 Hz and the intensity of 90 dB since the individual auditory threshold is not known. For both sides two consecutive measurement rounds with each consisting > 1000 stimulation responses were conducted. For analysis the latency of wave V (brainstem response) was used. The measurements were conducted in a low-noise environment to reduce external auditory stimuli. Subcutaneous needle electrodes were positioned above the processus mastoideus for recording. The quality of the resulting electrical potential was constantly monitored by a neurologist trained in neurophysiology.

### Collection of systemic vital parameters

During the experiment mean arterial pressure (MAP), heart rate (HR), oxygen saturation (SpO_2_) and respiratory rate (RR) were continuously monitored and recorded in patients. In the healthy subjects only SpO_2_, HR and RR but not MAP were monitored. The signals were acquired by means of Philips Intellivue system (Philips Medical systems, Boeblingen, Germany) that transferred the data to a CNS Data Collector (Moberg ICU Solutions, Ambler, Philadelphia, USA). We collected and exported the data from the CNS monitor using our proprietary data collection system ICU Cockpit. Signals were sampled at 1024 Hz.

### Processing of fNIRS-data and statistical analysis

Analyses were performed in MATLAB_R2019b (Mathworks, Natick MA, USA). Raw fNIRS signal O_2_Hb instead of deoxyhemoglobin (HHb) was used for the statistical analysis because of its better signal-to-noise-ratio^19,28^.

1. *SEP/AEP-fNIRS* Raw fNIRS data were set to zero-line and band-pass filtered between 0.01 and 0.2 Hz. This filtering removed physiological noise such as respiration and cardiac pulsation. The signal was segmented by the different conditions B1, S, B2, according to the time points registered during the experiment. For each segment, we calculated the distribution of spectral power between 0.01 and 0.2 Hz according to Welch’s method. We used the MATLAB program *pwelch* with the following parameters: high pass cutoff = 0.2 Hz, low pass cutoff = 0.01 Hz, filter order = 6, windows length = 60 × (sampling frequency = 25 Hz); samples overlap = 30; nfft = 2048. We first performed a qualitative analysis of power spectral distribution of healthy subjects during AEP and SEP. Analogously to our previous works^19,20^, changes in LFO were calculated through a ratio of mean spectral power within LFO between subsequent experimental blocks, i.e. from the first baseline to stimulation (B1 → S) and from stimulation to the second baseline (S → B2). The ratio was then transformed with the natural logarithm (*Ln*) as follows:
  $$LFO.RatioB1\to S=Ln\left( \frac{mean\left( LFO.PowerS \right)}{mean\left( LFO.PowerB1 \right)} \right),$$
  $$LFO.RatioS\to B2=Ln\left( \frac{mean\left( LFO.PowerB2 \right)}{mean\left( LFO.PowerS \right)} \right).$$
  The wilcoxon signed rank test was applied to compare LFO ratios of the ipsilateral and contralateral lobe.
2. *Resting-state recording* O_2_Hb time-series were set to zero-line and band-pass filtered between 0.01 and 4 Hz (filter order = 6, type = Butterworth). The filtering intentionally included physiological noise such as respiration and cardiac pulsation. The low pass filter of 4 Hz allowed also the inclusion of tachycardic patients. We defined the region of low frequency oscillations (LFO) within the band from 0.04 to 0.15 Hz^29–32^. For each subject we calculated the spectral power within the LFO (LFO.Power) region and normalized the value dividing it by the average spectral power within the frequency band 0.01–4 Hz (Totalpower). The normalized spectral power within LFO-region (LFO.n) was obtained according to the following equation:
  $$LFO.n=Ln\left( \frac{mean\left( LFO.Power \right)}{mean\left( Totalpower\right)} \right).$$
  LFOn obtained from the left and right hemisphere was averaged. Then we compared the values of LFOn between patients with clinical cerebral death and healthy subjects.
  For each subject average heart frequency and respiratory rate as long as its standard deviation were measured during the experiment. As additional confirmation, the peaks corresponding to the average heart frequency and respiration were qualitatively clearly visible in the spectral power analysis of fNIRS signal. Thus, we were able to calculate the spectral power within the frequency regions of heartbeat (Heartpow) and respiration (Respow) for each subject, analogously to the calculation of LFO by considering the frequency region between two standard deviations from the average frequency. The spectral power within these frequency bands was normalized dividing it by the average spectral power within the frequency band 0.01–4 Hz (Totalpower), according to the following equations:
  $$Heartpow.n=Ln\left( \frac{mean\left( Heart.power \right)}{mean\left( Totalpower\right)} \right),$$
  $$Respow.n=Ln\left( \frac{mean\left( Respow \right)}{mean\left( Totalpower\right)} \right).$$

Finally, we compared the spectral power within LFO region to Heartpow and Respow using the Wilcoxon rank sum test.

## Results

### FNIRS power spectrum analysis, qualitative assessment in the healthy subjects

**Figure 4:**
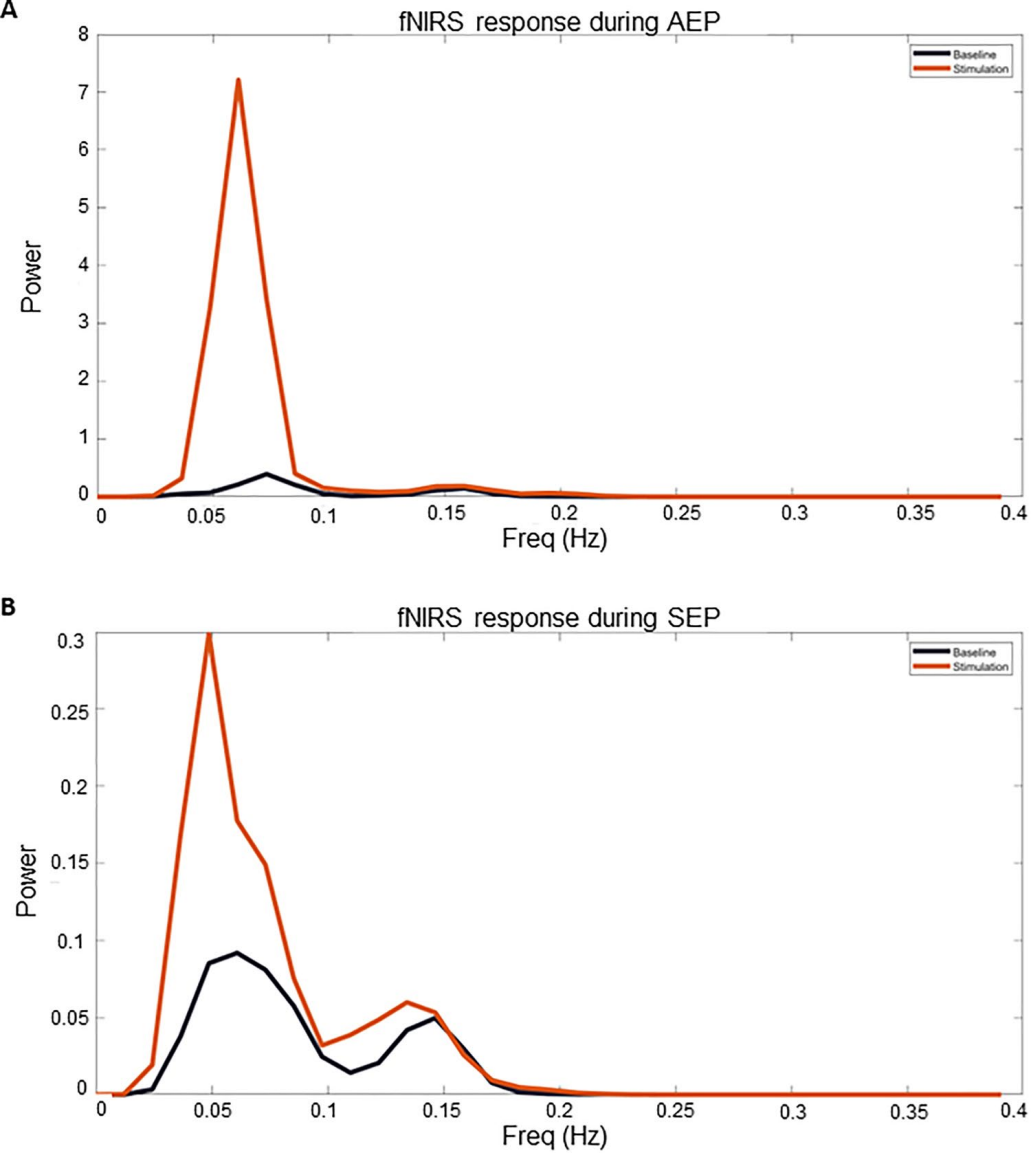
Spectral power analysis of two examples. (**A**) spectral power analysis of the fNIRS signal for O_2_Hb on the right hemisphere after repetitive acoustic stimulation on the right during a AEP (healthy subject 3). (**B**) spectral power analysis of the fNIRS signal for O_2_Hb on the right hemisphere after medianus stimulation on the left during a SEP (healthy subject 5).

The qualitative analysis of the power spectra of the fNIRS signal in the 7 healthy subjects shows a peak of the distribution of power in the region of LFO around 0.05–0.1 Hz. After exposure to somatosensory and acoustic stimulation, a common pattern of power increase in the frequency region of LFO could be identified. Two examples are given in Fig. 4.

### Measuring the cerebral response to AEP and SEP, quantitative approach in healthy subjects

#### AEP measurements in healthy subjects

**Figure 5:**
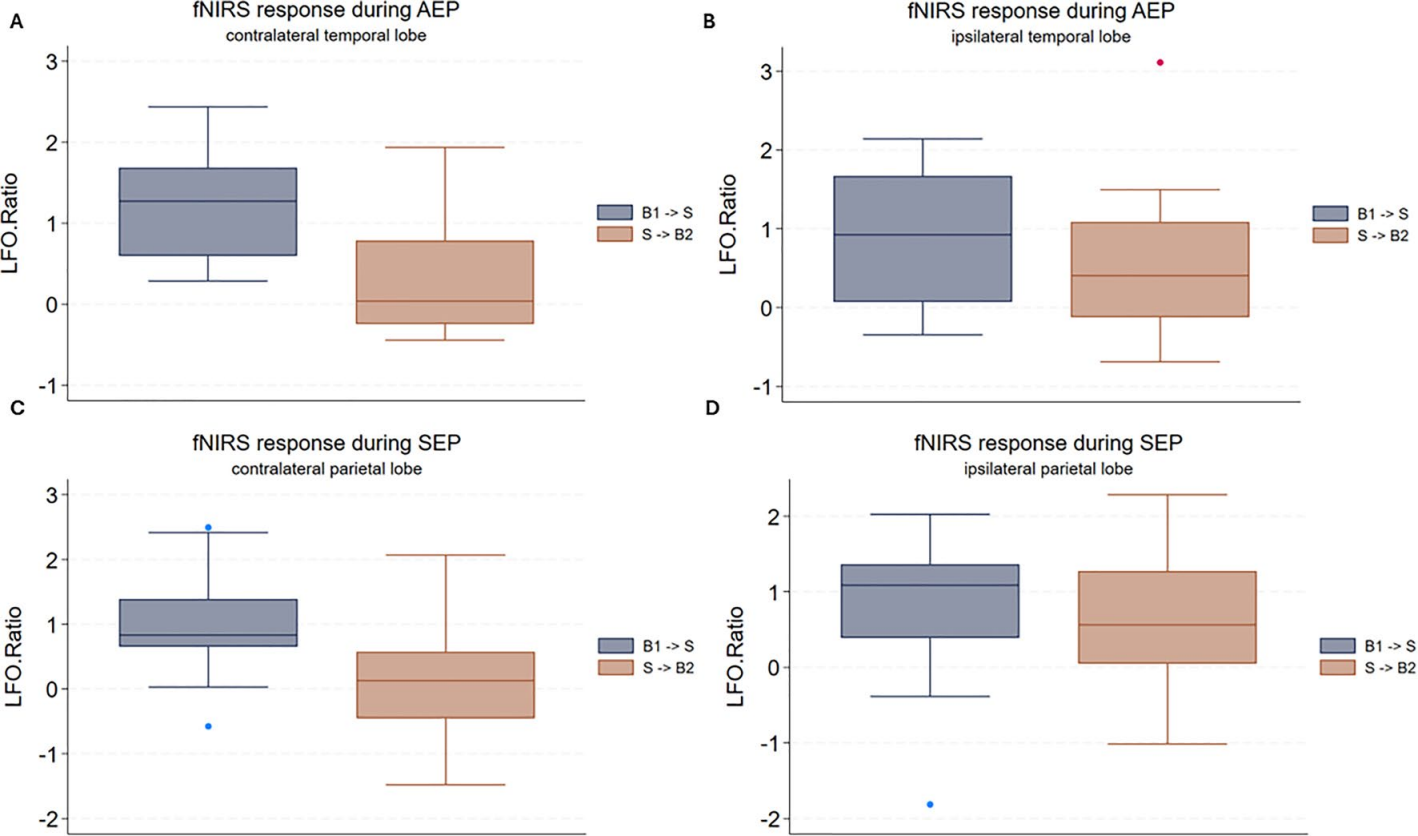
Boxplot analysis of functional near-infrared (fNRIS) response during auditory or somatosensory evoked potentials (AEP or SEP). The y-axis illustrates the ratio of low-frequency oscillation (LFO) power calculated between consecutive experimental blocks, while the x-axis denotes the sequential experimental blocks. B1 → S: from the initial baseline recording to sensory stimulation. S → B2: from sensory stimulation to the second baseline. (**A**) Boxplot of fNIRS response to acoustic evoked potential above the temporal lobe on the opposite side to stimulated ear. (**B**) Boxplot of fNIRS response to acoustic evoked potential above the temporal lobe on the same side to stimulated ear. (**C**) Boxplot of fNIRS response to somatosensory evoked potential above the parietal lobe on the opposite side to stimulated median nerve. (**D**) Boxplot of fNIRS response to somatosensory evoked potential above the parietal lobe on the same side to stimulated median nerve.

For each subject AEP registered a physiological electrical brain stem response with normal latencies of wave V(mean 5.9 ms, SD 0.2). The hemodynamic response measured by fNIRS is shown in Fig. 5. An increase of the spectral power in the LFO region was observed on both hemispheres after the acoustic stimulation, the increase was more evident on the contralateral temporal lobe. Comparing the two subsequent experimental blocks, LFO.Ratio_B1→S_ was significantly higher than LFO.Ratio_S→B2_ (Wilcoxon signed rank test, rank statistic = 271, p < 0.01) considering the contralateral temporal lobe, while no significant difference (Wilcoxon signed rank test, rank statistic = 244, p = 0.06) was found when considering the ipsilateral temporal lobe.

#### SEP in healthy subjects

One out of seven healthy controls had to be excluded from the SEP due the recording of pathologic N9 and P20 latencies. The other six, with a mean height of 180.8 cm (SD 6.3) were all within the limits of the laboratory defined reference values including one standard deviation. Mean P20 latencies were 21 ms (SD 1.3), Mean Inter Peak latencies MW I–V were 4.4 ms. For each run of SEP the fNIRS response on both hemisphere was measured. The hemodynamic response measured by fNIRS is shown in Fig. 5. An increase in LFO power after the medianus stimulation (B1 → S) couldn be observed on both hemispheres, however this effect was more evident on the contralateral hemisphere. After cessation of medianus stimulation (S → B2) no further increase in LFO power was observed. Comparing the two subsequent experimental blocks, LFO.Ratio_B1→S_ was significantly higher than LFO.Ratio_S→B2_ (Wilcoxon signed rank test, rank statistic = 271, p < 0.01) considering the contralateral parietal lobe. No significant difference between LFO.Ratio_B1→S_ and LFO.Ratio_S→B2_ was found when considering the ipsilateral parietal lobe (Wilcoxon signed rank test, rank statistic = 244, p = 0.06).

### Spectral power assessment of different frequency bands in patients with cerebral death: LFO, heart rate, respiratory rate

**Figure 6:**
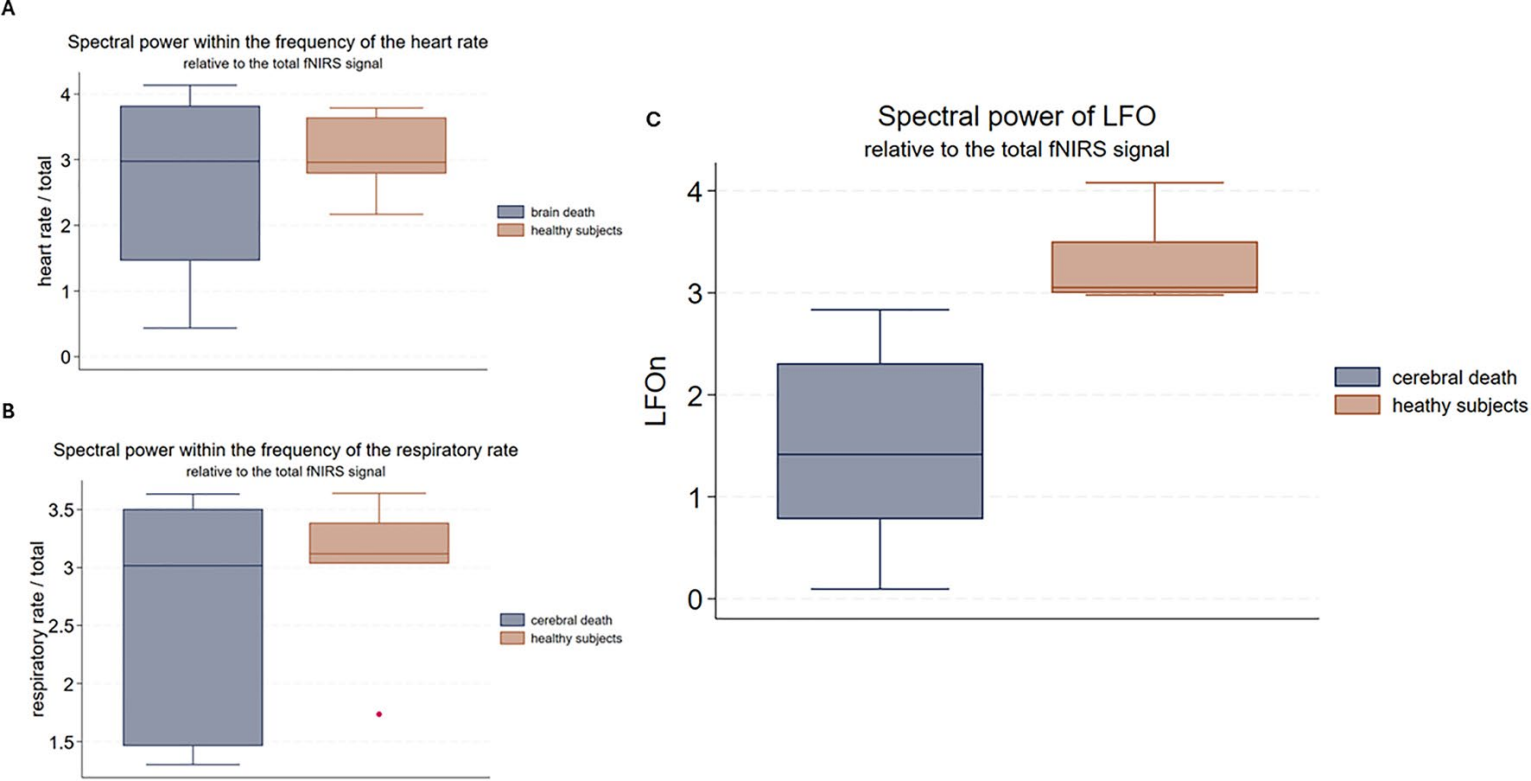
Boxplot analysis of spectral power in cerebral death patients and healthy subjects. (**A**) Normalized spectral power within the band of heartbeat in patients with cerebral death compared to healthy subjects. (**B**) Normalized spectral power within the respiratory rate band in patients with cerebral death compared to healthy subjects. (**C**) Normalized spectral power within the band of low frequency oscillations (LFOn) in patients with cerebral death compared to healthy subjects.

The normalized values of spectral power within the LFO region during the baseline recording was significantly lower in the patients with cerebral death (n = 7) as compared with the 6 healthy subjects (Wilcoxon ranksum 28, p = 0.001). Patients with cerebral death and healthy subjects did not show significant differences in spectral power for physiological activities such as heartbeat and respiration rate (Heartpow.n: Wilcoxon ranksum 48, p = 0.98; Respow.n: Wilcoxon ranksum 57, p = 0.29). The results are presented in the Fig. 6.

## Discussion

This study was aimed to gain better understanding on the oscillatory hemodynamic signal within the spectral region of LFO measured by fNIRS. First, in healthy subjects with physiological electrical response following both sensory and acoustic stimulation (SEP/AEP) our study showed an increase in the spectral power of LFO predominantly localized in the contralateral parietal and temporal area respectively. This finding is consistent with the commonly known crossed neuroanatomical arrangement of the ascending sensory and auditory pathways. Second, patients after cerebral death showed remarkably lower values of spectral power within LFO region in comparison to healthy subjects during resting-state fNIRS recordings. On the contrary, patients after cerebral death exhibited no significant difference when compared to healthy subjects in other pertinent spectral regions that reflect systemic physiological activities, such as respiration and heartbeat.

Far from being an exclusive fNIRS biomarker, LFO around 0.1 Hz have been widely described by research work with functional magnetic resonance and are thought to reflect local vasomotor response to neuronal activity^29,33–35^, eventually modulated by the sympathetic nervous system^36^. The mutual influence of neuronal electrochemical activity and local hemodynamic is generally referred to as neurovascular coupling^37^. However, the way these elements interact (e.g. according to EEG-fMRI based research protocols) is still poorly understood^38,39^. Further on, LFO spectral region certainly contains extracerebral derived signal, which is hard to separate from the neurogenic one^40–42^. The extracerebral signal is probably originated by systemic blood pressure oscillation, including Mayer waves^43^, under the influence of autonomic nervous system. Our research brings solid evidence of LFO centered hemodynamic response, mirroring the electrical evoked potentials, which can be detected by fNIRS, consistent to the hypothesis of an underlying neurovascular coupling.

Further on, our results showing a strongly reduction of LFO in the patients after cerebral death strongly reinforces the assumption that LFO reflects a specific neurogenic hemodynamic activity and may be used as fNIRS-biomarker for cortical responsivity. In contrast to LFO, heartbeat and respiration was still detectable in the fNIRS recordings after cerebral death, probably because of an intrusion of extracranial hemodynamic activity. After cerebral death, we expect fNIRS to exclusively detect extracranial hemodynamic activity. As anticipated, fNIRS recordings in patients with cerebral death displayed normal spectral power within the frequencies linked to heartbeat and respiration, while spectral power of LFO was strongly diminished in comparison to heathy subjects.

Changes in O2Hb levels after exteroceptive stimulation have been already extensively studied across various scenarios, including voluntary movement, auditory stimulation^44^, peripheral nerve stimulation^45^, and painful sensory stimulation^46^. In the last years, few studies on brain-computer interfaces pioneered the exploration of neurovascular coupling with simultaneous fNIRS and electrophysiological measurements^47–52^. In fact, incorporating hemodynamic response alongside electrical response to sensory stimuli in the cerebral cortex could enhance the accuracy of brain activity assessment. Prior research has primarily focused on analyzing changes in concentration of O2Hb, HHb or total hemoglobin. Conversely, our study innovatively employs a signal frequency-based methodology. Moreover, this is the first study using established AEP and SEP measurement protocols as it is used in clinical practice rather than designing specific acoustic or sensory stimulation protocols.

Further on, we suggest LFO as new hemodynamic biomarker to evaluate cortical responsivity in patients with suspected cessation of brain activity such as by brain death. Prior fNIRS studies have suggested a multi-phase protocol at different fractions of inspired oxygen, administered through the ventilator, to evaluate brain death through alterations in the concentration ratios of O2Hb and HHb^53,54^. Assessing LFO during resting-state could serve as a rapid bedside method without necessitating additional intervention in ventilator settings.

Nevertheless, certain limitations should be considered when interpreting our findings. Firstly, as this is an exploratory study, the results should be validated with a larger experimental sample. Secondly, even if the age difference between the groups is not statistically relevant, a reduction in LFO associated to age-related neurodegeneration has been described previously and may have, to some minor extent, influenced the results^55–57^. Additionally, as cortical auditory evoked potentials are not established in our center on a regular base we decided against utilising wave VI and VII for analysis. Therefore AEP monitoring was only carried out as far as the inferior colliculus (wave V) and the direct comparability to the cortical fNIRS measurement is limited by its regional difference. Further on, since only healthy subjects were measured with AEP/SEP, more studies are needed to understand if fNIRS performs as well as standard electrical evoked potential in the detection of cortical activation, especially in patients with disorders of consciousness. Last, it is important to highlight that our observation of diminished LFO following cerebral death is purely descriptive. Further investigations are necessary to evaluate the reliability of LFO in resting-state recordings for assessing the absence of cerebral perfusion.

## Conclusion

Overall, our results provide evidence of the specifically neurogenic origin of LFO and of its coupling with electrical brain activation. LFO is a promising fNIRS biomarker for assessment of cerebral reactivity.

## Data Availability

Raw data were generated in the neurocritical care unit of the university hospital of Zurich. The datasets generated and analysed during this study are available from the corresponding author upon request.
